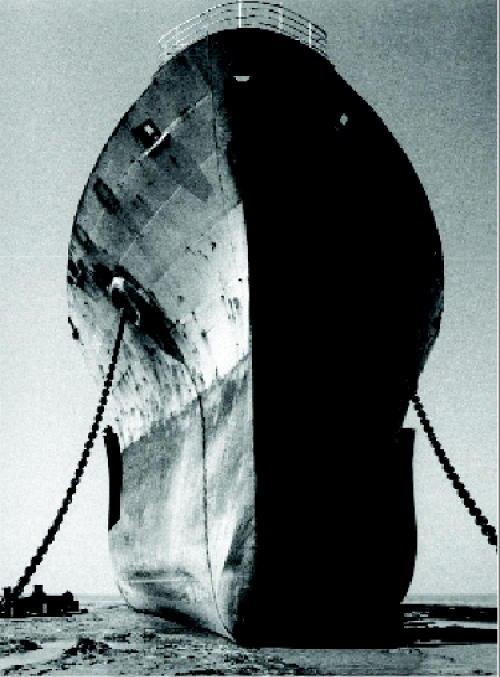# The Beat

**Published:** 2005-03

**Authors:** Erin E. Dooley

## LAX Pays Its Neighbors Back

A first-of-its-kind community benefits agreement was signed in December 2004, providing a benefits package worth millions in coming years to residents living near Los Angeles International Airport. The airport, the second largest industrial smog source in the Los Angeles area, currently supports about 1,000 flights each day, and a major expansion project is in the works. The deal includes monies for soundproofing of schools and homes, and encourages green building and energy conservation practices. The agreement also calls for studies on the sources and health risks of toxic air emissions from local sources, including the airport.

## Cookstove Monitors Tested in Honduras

Handmade wood-fueled cookstoves are widely used throughout the developing world. The smoky stoves produce large amounts of particulate matter inside the home, affecting the health of everyone living there, especially women and children, who spend the most time indoors. Now a team from the University of Illinois at Urbana–Champaign has designed a portable, battery-operated sampling cart to measure emissions in remote locations. The cart has sensors for measuring carbon dioxide and carbon monoxide, instruments for measuring particle color and concentration, and filters for collecting particles for later analysis. Onsite sampling lets researchers see how actual cooking practices (such as the type of wood used) affect stove outputs. The carts have been used in a baseline study in Honduras and will be used again in summer 2005 to measure emissions from new, more efficient stoves that have been distributed.

## Watch Out, Worms!

What do colorless, see-through, tube-shaped organisms made up of only 959 cells have in common with laboratory rats . . . and with humans? A new five-year, $4 million study at Duke University funded by the National Toxicology Program seeks to determine just that. *Caenorhabditis elegans*, the subject of the Duke study, lives in soils around the world, where it feeds on bacteria. The worm has already been extensively studied, but this project will focus on developing rapid toxicologic assays using the organism. Such assays should help lower the costs of and number of rodents needed for carrying out a typical toxicology assay.

## Overfishing and Bushmeat

Companies from the European Union fish heavily off the West African coast, with financial subsidies for fleets topping $350 million in 2001. Now a report published 12 November 2004 in *Science* states that declining fish stocks—down by at least 50% since 1970—are forcing local peoples to slaughter wildlife, or “bushmeat,” for food. Over the same time period, this demand for bushmeat has led to a 76% decline in the numbers of 41 species of mammals, including buffalo, antelope, jackals, monkeys, and elephants; several species are now near extinction. The study group states that measures are urgently needed to help local peoples find cheap and readily available alternative protein sources. Furthermore, until larger issues such as international fish export agreements are addressed, local efforts to prevent wildlife extinction will fall short.

## Counting Sea Creatures

It is believed that only 5% of the world’s oceans have been explored so far, but new technologies are opening up new areas of the underwater world every day. In November scientists announced they had discovered 106 new species of fish in 2004 alone as part of the 10-year Census of Marine Life, begun in 2000. More than 1,000 scientists from 70 countries are taking part in the census. Facilitating the exchange of this new information is a publicly accessible database (http://www.iobis.org/). The census database has more than 5.2 million records mapping the distribution of 38,000 marine species, a significant increase from the 1.1 million records of 25,000 species last year. To date about 230,000 marine species have been described by scientists. Census members believe the actual number of species may be 10 times that.

## Anchoring Toxic “Ghost Ships”

The parties to the Basel Convention affirmed in October 2004 that aging oceangoing ships, often called “ghost ships,” are to be considered toxic waste under international law, meaning they cannot be exported for dismantling. Until now such ships were often sent to countries such as India, Bangladesh, Pakistan, Turkey, and China, where laborers would break down the vessels to recover valuable steel. The ships often contain asbestos, PCBs, mercury, lead, waste fuel oil, and other toxic substances that contaminate the areas around the shipbreaking yards. The convention now has asked its Open-Ended Working Group to consider a legally binding ban on the movement of such ships to be voted on at the convention’s 2005 assembly.

## Figures and Tables

**Figure:**
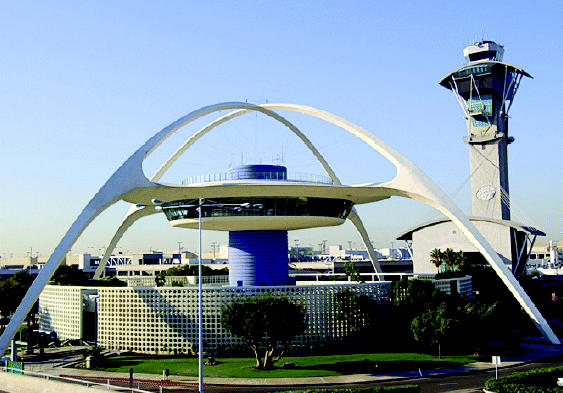


**Figure:**
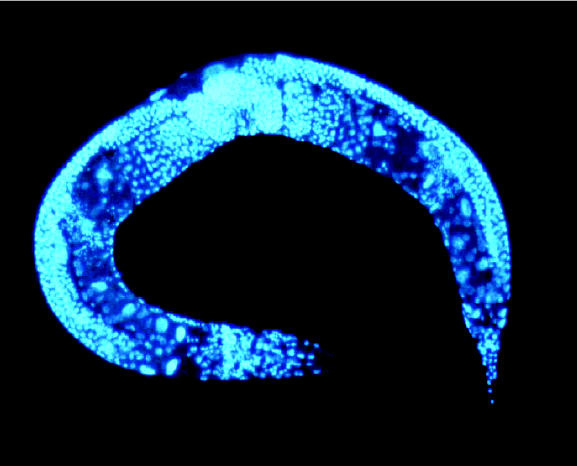


**Figure:**
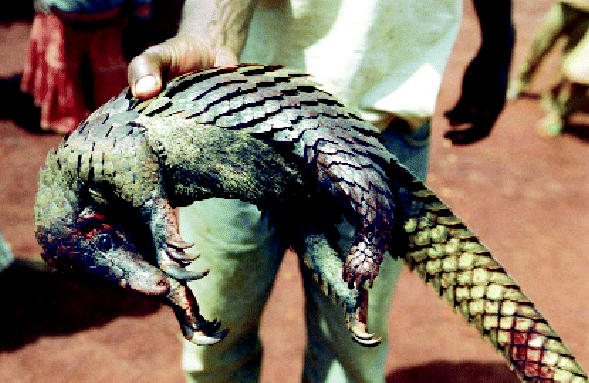


**Figure:**